# Bullous Pemphigoid and Diabetes medications: A disproportionality analysis based on the FDA Adverse Event Reporting System

**DOI:** 10.7150/ijms.55421

**Published:** 2021-03-03

**Authors:** Liting Huang, Ying Liu, Huijun Li, Weicun Huang, Ruirui Geng, Zaixiang Tang, Yiguo Jiang

**Affiliations:** 1 Department of Biostatistics, School of Public Health, Medical College of Soochow University, Suzhou, 215123, China.; 2 Jiangsu Key Laboratory of Preventive and Translational Medicine for Geriatric Diseases, Medical College of Soochow University, Suzhou, 215123, China.; 3 National Institute for Food and Drug Control, Beijing, 102629, China.; 4 Department of pharmacy, The Affiliated Suzhou Science & Technology Town Hospital of Nanjing Medical University, Suzhou, 215153, China.

**Keywords:** diabetes medications, dipeptidyl peptidase 4 inhibitors, Bullous Pemphigoid, FAERS, drug safety

## Abstract

**Background:** The world's first Diabetes Medications (Insulin) was marketed in October 1923. Some studies suggested the association of diabetes medications with Bullous Pemphigoid (BP), especially the Dipeptidyl Peptidase 4 (DPP-4) inhibitors. The study aims to detect an association between diabetes medications (focusing on DPP-4 inhibitors) and bullous pemphigoid based on FDA Adverse Event Reporting System (FAERS).

**Methods:** All spontaneous reports of diabetes medications inhibitors-related BP recorded in the FAERS between March 2004 and August 2020 were included in the present study. Disproportionality analysis was performed to find the signal between diabetes medications and BP. The Chi-Squared with Yates' correction (χ^2^_Yates_), proportional reporting ratio (PRR) and the lower limit of the 95% confidence interval of the Reporting Odds Ratio (ROR_025_) were calculated as a measure. A signal was detected when ROR_025_ > 1, PRR > 2, χ^2^_Yates_ > 4 and at least 3 cases.

**Results:** There were 3770 reports for BP in FAERS. The strongest signal for diabetes medications-BP association were DDP-4 inhibitors (ROR_025_: 13.700, PRR: 15.408), followed by Meglitinides (ROR_025_: 12.708, PRR: 16.777), Non-sulfonylureas (ROR_025_: 6.434, PRR: 7.016), Alpha-glucosidase inhibitors (ROR_025_: 6.105, PRR: 10.738), Sulfonylureas (ROR_025_:2.655, PRR: 3.200).

**Conclusions:** This study detected a strong signal between BP and DDP-4 inhibitors, alpha-glucosidase inhibitors, meglitinides, non-sulfonylureas, and sulfonylureas in FAERS. The signal was significantly higher with alogliptin than with the other DPP-4 inhibitors. The study doesn't suggest the association between the incretin mimetics, insulin, SGLT-2 inhibitors, thiazolidinediones and BP in FAERS.

## Introduction

Bullous pemphigoid (BP) is a rare acquired autoimmune skin condition. It usually develops on areas of skin that often flex, such as the lower abdomen, upper thighs, or armpits. The clinical manifestations of BP include tense bullae, urticarial skin lesions and pruritus, oral mucous membrane erosions that may be present in 10-20% of patients 1-3. In some patients, eczema-like erythema may proceed for months or even for many years as a prodromal phase before BP develops 1. A retrospective monocentric cohort study confirmed that BP was associated with high mortality 4. BP is most common in older adults, the incidence of BP appears to be equal in men and women and no known ethnic or racial predilection is detected for developing bullous pemphigoid 5. BP is caused by an autoimmune reaction against bullous pemphigoid antigen 180 (BP180) and/or bullous pemphigoid antigen 230 (BP230), both BP180 and BP230 are a major structural component of hemidesmosomes 6, 7. BP230 localizes intracellularly and associates with the hemidesmosomal plaque, BP180 is a transmembrane glycoprotein with an extracellular domain 6. Antibodies against both BP180 and BP230, as measured by ELISA, are used for the diagnosis of bullous pemphigoid 3. But the exact reason for this abnormal immune response is unknown, although it sometimes can be triggered by taking certain medications, trauma, burns, radiotherapy, ultraviolet irradiation, the phenomenon of epitope spreading or genetic factor 8, 9. There are more than 50 medications have been associated with BP development 10.

The world's first Diabetes Medications (Insulin) was marketed in October 1923. A study suggested the association of diabetes mellitus with BP 11. Meanwhile, DPP-4 inhibitors (also known as “gliptins”) and tolbutamide were associated with BP in the literature 10. So, it was necessary to analyze the association between diabetes medications and BP. For individual diabetes medications, non-sulfonylureas (including the metformin) and DPP-4 inhibitors should be focused on. Metformin was a classic antihyperglycemic drug and the top treatment choice for type 2 diabetes. Metformin always was used in combination with DPP-4 inhibitors. DPP-4 inhibitors are a class of diabetes medications that are used with diet and exercise to control high blood sugar in adults with type 2 diabetes. DPP-4 inhibitors lower blood sugar by helping the body increase the level of the hormone insulin after meals. Insulin helps move sugar from the blood into the tissues, so the body can use the sugar to produce energy and keep blood sugar levels stable. The DPP-4 inhibitors may induce anti-basement membrane zone antibodies or other structurally close antibodies 12, leading to BP. Inhibition of DPP-4 has been shown to enhance the recruitment of eosinophils into the dermis, which may contribute to the blister formation and tissue damage observed in BP 13. The inhibition of gliptins may cause the activation of eosinophils by a CCL11/eotaxin-mediated mechanism. The activation of eosinophils and lymphocyte infiltration substantially contributes to the appearance of blisters and tissue damage in bullous pemphigoid. On the other hand, DPP-4 inhibitors may alter the antigenic properties of the epidermal basement membrane 14. Even though an increasing number of cases of BP induced by DPP-4 inhibitor was reported in the literature, the exact mechanism underlying this association remains unclear and needs to be elucidated 8.

Previously, some case reports supported the hypothesis that there is a risk of BP in patients exposed to DPP-4 inhibitors 12, 14-20. Some retrospective studies suggested that the use of DPP-4 inhibitors is associated with the development of BP in patients with diabetes 21, 22. A meta-analysis suggested that DPP-4 inhibitor exposure is associated with a significantly increased risk for BP 23. And the warnings and precautions of DPP-4 inhibitors' latest label in the FDA showed that there have been reports of bullous pemphigoid requiring hospitalization. But other types of diabetes medications' labels in the FDA didn't include the warnings about bullous pemphigoid.

Data mining algorithms (DMAs) are currently and routinely used by pharmacovigilance experts for quantitative signal detection 24. The accuracy of data mining techniques has been already tested retrospectively to determine if already known safety issues would have been detected 'earlier' 25. Some scholars conducted disproportionality analyses based on DMAs for all spontaneous reports from the French, European, Japanese, WHO and Spanish Pharmacovigilance Database 8, 9, 26-28. These studies based on the pharmacovigilance databases all showed a significant association between DPP-4 inhibitors and BP.

FDA Adverse Event Reporting System (FAERS) was the pharmacovigilance database of the United States. We investigated the association between all types of diabetes medications (focused on DPP-4 inhibitors) and BP using the data from FAERS based on DMAs in this study. In addition, the pooled analysis based on DMAs between the DPP-4 inhibitors and BP was made by combining French, American, Japanese, WHO and Spanish Pharmacovigilance Database in the study.

## Materials and Methods

### Study Design

A retrospective analysis was conducted to comparatively assess BP reports with Diabetes Medications. Acetaminophen was considered as a negative control, whereas furosemide illustrated descriptive positive control 9, 10.

### Data source

Data in the present study were obtained from the public release of the OpenVigil FDA (https://openvigil.pharmacology.uni-kiel.de/openvigilfda.php), which covers the period from March 2004 through August 2020 in the FAERS.

The data currently used in OpenVigil FDA was obtained from FAERS 29, 30. OpenVigil FDA is a pharmacovigilance tool to extract and analyze FAERS data using the OpenFDA API for accessing the FDA drug-event-database with the additional OpenFDA drug mapping and duplicate detection functionality, OpenFDA aims at providing clean and curated access to the underlying AERS and can count reports stratified to an extraction condition 29, and it overcame some disadvantages of FAERS.

In the study, DPP-4 inhibitors were limited to the approved drugs by the FDA (sitagliptin, saxagliptin, linagliptin, alogliptin). The study analyzed the pooled DPP-4 inhibitors and each DPP-4 inhibitor individually. For reducing the interference from gender, this study also analyzed the pooled DPP-4 inhibitors and each DPP-4 inhibitor individually by a different gender.

Most patients with DDP-4 inhibitors received combinations of other medications. A sensitivity analysis was made after excluding cases where drugs other than DPP-4 inhibitors were suspected in the BP occurrence (Supplementary Table 1) to reduce the confounding bias.

Diabetes medications other than DPP-4 inhibitors analyzed in the study were listed in Supplementary Table 2. The study also analyzed the association between diabetes medications and BP after excluding the cases of combined use of DPP-4 inhibitors to reduce the DDP-4 inhibitors' interference.

### Definition of adverse events

Adverse events in the OpenVigil FDA were coded according to the terminology preferred by the Medical Dictionary for Regulatory Activities (MedDRA) Preferred Terms (PTs). For the disproportionality analysis, pemphigoid (PT10034277) were selected for mining according to the MedDRA 22.0.

### Data mining algorithms

Data mining algorithms (DMAs) can be classified in the frequentist and Bayesian approach. The frequentist methods are based on the same principles of calculation using the 2×2 table (Supplementary Table 3) 31. The study calculated proportional reporting ratio (PRR), Reporting Odds Ratio (ROR), ROR_025_, and Chi-Squared with Yates' correction (χ^2^_Yates_) based on the frequentist approach from adverse drug reaction reports determining whether the combination of drug and adverse event are related.

These values were calculated on the Open Vigil-2×2 contingency table calculator (https://openvigil.pharmacology.uni-kiel.de/contingency-table-calculator.php) in the study.

For the study, when PRR > 2, χ^2^_Yates_ > 4 (= p < 0.05), the lower limit of the 95% confidence interval of the ROR (ROR_025_) is greater than one and at least 3 cases as minimal criteria for a signal of disproportionality 31, 32.

## Results

### Case selection

During the study period (between 2004 and 2020), 12254196 adverse drug reaction reports were entered in the OpenVigil FDA. Among these, 89277 adverse drug reaction reports were related to DPP-4 inhibitors, and 3770 adverse drug reaction reports were related to BP. Among these DPP-4 inhibitors' reports, 383 reports were related to BP (alogliptin, n = 70; linagliptin, n = 51; sitagliptin, n = 250; saxagliptin, n = 17), 5 of them involved two or more DPP-4 inhibitors.

### Characteristics of the DDP-4 inhibitors and control group

For the gender, the reaction tended to be more common in male (50.91%, 61.43%, 66.67%, 44.40% and 35.29% of pooled DPP-4 inhibitors-, alogliptin-, linagliptin-, sitagliptin-, saxagliptin-related cases, respectively) and elderly people-at least 75 years (52.22%, 68.57%, 35.29%, 49.20% and 70.59% of pooled DPP-4 inhibitors-, alogliptin-, linagliptin-, sitagliptin-, saxagliptin-related cases, respectively). For the control group, the gender distribution is different, acetaminophen-related cases tended to be more common in female (58.33%), but furosemide-related cases tended to be more common in male (52.58%). The entire control group tended to be elderly people-at least 75 years (45.00% and 55.32% of acetaminophen- and furosemide-related cases, respectively). The age distribution of these cases was similar to the general BP population, but the gender distribution was different from the general BP population 5. The characteristics of DDP-4 inhibitors and the control group were summarized in Table 1.

### BP and DDP-4 inhibitors in the FAERS

The study made a general disproportionality analysis between DDP-4 inhibitors and BP in the FAERS. BP cases were reported more frequently for DPP-4 inhibitors than for the control group. For the DMAs result between pooled DDP-4 inhibitors and BP, it showed a signal, with the ROR_025_, PRR, the number of adverse events and χ^2^_Yates_ of 13.916, 15.408, 383, and 4624.373, respectively. For the DMAs result between the furosemide and BP, it showed a signal, with the ROR_025_, PRR, the number of adverse events and χ^2^_Yates_ of 3.838, 4.294, 329, and 756.041, respectively. For the DMAs result between the acetaminophen and BP, it didn't show a signal, with the ROR_025_, PRR, the number of adverse events and χ^2^_Yates_ of 0.418, 0.540, 60, and 22.657, respectively. For the DMAs result between each DDP-4 inhibitor and BP, it showed a signal. The largest disproportionality corresponded to alogliptin, followed in decreasing order by linagliptin, sitagliptin, and saxagliptin. The DMAs result between pooled DDP-4 inhibitors and BP in the gender did not suggest the different disproportionality result between the male and female. The results were summarized in Table 2.

The study also made a sensitivity disproportionality analysis between DDP-4 inhibitors and BP in the FAERS. For the DMAs result between pooled DDP-4 inhibitors and BP, it showed a signal, with the ROR_025_, PRR, the number of adverse events and χ^2^_Yates_ of 13.700, 15.362, 298, and 3672.735, respectively. The analysis values were different from the general disproportionality analysis, but it also displayed high disproportionality regarding the association between pooled DDP-4 inhibitors and BP. For individual DPP-4 inhibitors, the disproportionality order was the same as in the general disproportionality analysis. The results were summarized in Table 2.

### BP and DDP-4 inhibitors in the Pooled databases

By combining the results of the study with those previous studies conducted over the FPVD (France), JADER (Japan), FEDRA (Spanish) and VigiBase (WHO) databases 8, 9, 26, 27. For the DMAs result between DDP-4 inhibitors and BP in the Pooled databases, it showed a signal, with the ROR_025_, PRR, the number of adverse events and χ^2^_Yates_ of 60.276, 62.711, 1932, and 87122.550, respectively (Table 3).

### BP and other diabetes medications in the FAERS

For the DMAs result between the non-sulfonylureas and BP, it showed a signal, with the ROR_025_, PRR, the number of adverse events and χ^2^_Yates_ of 6.434, 7.016, 584, and 2541.646, respectively. After excluding case subjects who received DPP-4 inhibitors to reduce the interference of DPP-4 inhibitors, significant disproportionality did not disappear for case subjects receiving the non-sulfonylureas. For the DMAs result between the other individual diabetes medications and BP, the alpha-glucosidase inhibitors, meglitinides and sulfonylureas showed disproportionality regardless of whether excluding case subjects who received DPP-4 inhibitors, but the incretin mimetics (also known as GLP-1 Agonists), insulin, SGLT-2 inhibitors and thiazolidinediones did not show disproportionality regardless of whether excluding case subjects who received DPP-4 inhibitors. The OpenVigil FDA did not receive the report between amylin analogs and BP. These results were summarized in Table 4.

## Discussion

The DPP-4 inhibitors-related BP cases tended to be more common in males (presumably because DPP-4 inhibitors were used more often in males than in females 27) and elderly people (at least 75 years). The effect of DPP-4 inhibitors on BP did not have a statistical difference in gender in the FAERS. It was different from the result of a hospital-based Swiss-French study and a Finnish nationwide registry study, which found that the effect of DPP-4 inhibitors on BP had a statistical difference in gender 33, 34.

These results showed disproportionality for BP and DPP-4 inhibitors in the entire pharmacological databases and the FAERS regardless of whether excluding cases where drugs other than DPP-4 inhibitors were suspected in the BP occurrence, which was consistent with those reported in previous studies conducted in other countries' pharmacovigilance databases 8, 9, 26-28. Analysis of each DPP-4 inhibitor separately also showed a significant association. Alogliptin showed higher ROR_025_ than other DPP-4 inhibitors, followed in decreasing order by linagliptin, sitagliptin and saxagliptin. It was different from the previous studies 8, 9, 26-28, presumably because the different regulatory Agencies approved the different DPP-4 inhibitors. For example, the FDA did not approve the vildagliptin, which appeared a higher risk than the others in other countries' pharmacovigilance databases' study 9, 26-28. It was interesting to specify that sitagliptin was the most prescribed DPP-4 inhibitor in the USA 35. However, disproportionality analyses confirmed a higher risk in alogliptin. No clear reason has been found to explain the higher association of alogliptin with the development of BP compared with the other DPP-4 inhibitors. For negative control (acetaminophen), the study did not show disproportionality. For the positive control (furosemide), the study showed disproportionality. The results of the control group were consistent with those reported in previous studies 9, 10.

The alpha-glucosidase inhibitors, meglitinides, non-sulfonylureas and sulfonylureas with BP showed disproportionality regardless of whether excluding case subjects who received DPP-4 inhibitors. It was different from the results of the JADER database and Finnish nationwide case-control study at Rambam Health Care Campus, Haifa, Israel 27, 36. The incretin mimetics, insulin, SGLT-2 inhibitors and thiazolidinediones with BP did not show disproportionality regardless of whether excluding case subjects who received DPP-4 inhibitors. The above results were different from the results of the JADER database, which showed that the significant ROR disappeared for case subjects receiving the other individual diabetes medications after excluding case subjects who received DPP-4 inhibitors 27. For the alpha-glucosidase inhibitors, meglitinides, non-sulfonylureas, and sulfonylureas, perhaps because the FAERS database had more reports than the JADER database or the association of diabetes mellitus with BP. In those early reports, the association of diabetes mellitus with BP had been analyzed, and possible underlying mechanisms that increased skin fragility due to elevated glucose levels and the induction of autoantibody production by glycosylation of dermal proteins were suggested 11. The DMAs results between other individual diabetes medications and BP did not change after excluding case subjects who received DPP-4 inhibitors. It meant that maybe other types of diabetes medications did not interact with DPP-4 inhibitors on BP.

Our study has limitations. The FAERS database was a spontaneous reporting system rather than a mandatory reporting system, the reporters consisted of patients, caregivers, and manufacturers. FDA did not receive reports for every adverse event or medication error that occurs with a product. This introduced an inevitable selection bias, and reporting biases may be differential across different drugs. There was no specific role to check the data in the report, the entry errors couldn't be controlled, such as typographical errors and spelling mistakes.

Moreover, concomitantly administered drugs, age groups and indications possibly introduced confounding bias. To exclude this possible effect, a sensitivity analysis that excluded the cases where drugs other than DPP-4 inhibitors were suspected in the BP occurrence had been made in the study, but BP events that may be caused by unknown drugs' interactions hadn't been excluded. And the patients' other concomitant diseases or drugs or indications were limits in the FAERS report.

Additionally, the FDA did not require that a causal relationship between a product and event be proven, and reports did not always contain enough detail to properly evaluate an event. Mapping names of pharmaceutical products to an active substance is still not sufficiently resolved the issue in pharmacovigilance and epidemiology 37. So we can use this database to generate hypotheses rather than hypotheses testing, the database can't be used to calculate the incidence of an adverse event or medication error in the United States or establish any causal relationship.

In general, further study, particularly clinical trials, is required with better data sources and research design to ensure whether Diabetes Medications have any synergistic effect on BP.

## Conclusion

In conclusion, this study suggests a strong signal between bullous pemphigoid and DDP-4 inhibitors in the FAERS and the combining data from French, Japanese, WHO, Spanish and American pharmacovigilance databases. The signal was significantly higher with alogliptin than with the other DPP-4 inhibitors in the FAERS. The effect of DPP-4 inhibitors on BP did not have a statistical difference between gender in the FAERS.

The study also suggests the association between alpha-glucosidase inhibitors, meglitinides, non-sulfonylureas, sulfonylureas and BP in the FAERS. And it doesn't suggest the association between the incretin mimetics, insulin, SGLT-2 inhibitors, thiazolidinediones and BP in the FAERS.

## Supplementary Material

Supplementary table 1.

Supplementary table 2.

Supplementary table 3.

## Figures and Tables

**Table 1:** General characteristics of cases of bullous pemphigoid associated with DDP-4 inhibitors and the control group in FAERS

	Gliptins	Alogliptin	Linagliptin	Sitagliptin	Saxagliptin	Acetaminophen	Furosemide
**Gender**							
Female	154 (40.21%)	21 (30.00%)	12 (23.53%)	117 (46.80%)	10 (58.82%)	35 (58.33%)	135 (41.03%)
Male	195 (50.91%)	43 (61.43%)	34 (66.67%)	111 (44.40%)	6 (35.29%)	23 (38.33%)	173 (52.58%)
UK	34 (8.88%)	6 (8.57%)	5 (9.80%)	22 (8.80%)	1 (5.88%)	2 (3.33%)	1 (0.30%)
**Age**							
≤44	7 (1.83%)	2 (2.86%)	3 (5.88%)	2 (0.80%)	0 (0.00%)	2 (3.33%)	1 (0.30%)
45-64	33 (8.62%)	6 (8.57%)	4 (7.84%)	24 (9.60%)	1 (5.88%)	13 (21.67%)	31 (9.42%)
65-74	82 (21.41%)	7 (10.00%)	15 (29.41%)	60 (24.00%)	1 (5.88%)	12 (20.00%)	80 (24.32%)
≥75	200 (52.22%)	48 (68.57%)	18 (35.29%)	123 (49.20%)	12 (70.59%)	27 (45.00%)	182 (55.32%)
UK	61 (15.93%)	7 (10.00%)	11 (21.57%)	41 (16.40%)	3 (17.65%)	6 (10.00%)	35 (10.64%)
Total	383	70	51	250	17	60	329

**Table 2:** The general and sensitivity DMAs results between DDP-4 inhibitors/control group and bullous pemphigoid

Drugs	a	χ^2^_Yates_	PRR	ROR	ROR_025_
***Gliptins***	383	4624.373	15.408	15.470	13.916
Male	195	1830.141	12.454	12.511	10.783
Female	154	2094.144	17.102	17.161	14.529
Sensitivity analysis result	298	3672.735	15.362	15.425	13.700
***Alogliptin***	70	6065.722	91.544	94.118	74.054
Male	43	3069.234	76.767	79.363	58.359
Female	21	1707.185	88.469	90.300	58.449
Sensitivity analysis result	58	5396.474	98.125	101.101	77.701
***Linagliptin***	51	649.065	15.107	15.172	11.501
Male	34	460.425	16.168	16.276	11.580
Female	12	82.490	9.513	9.532	5.398
Sensitivity analysis result	39	526.123	15.945	16.018	11.675
***Sitagliptin***	250	2535.917	12.830	12.874	11.322
Male	111	733.032	8.979	9.009	7.434
Female	117	1593.627	16.776	16.834	13.941
Sensitivity analysis result	191	1923.614	12.587	12.630	10.916
***Saxagliptin***	17	68.786	6.203	6.213	3.856
Male	6	8.287	3.489	3.493	1.566
Female	10	74.318	10.283	10.305	5.530
Sensitivity analysis result	14	61.864	6.682	6.694	3.958
***Acetaminophen***	60	22.657	0.540	0.540	0.418
Male	35	3.018	0.735	0.735	0.526
Female	23	12.803	0.473	0.473	0.313
Without Gliptins	59	22.546	0.538	0.538	0.416
***Furosemide***	329	756.041	4.294	4.298	3.838
Male	135	148.620	2.849	2.852	2.393
Female	173	597.785	5.730	5.736	4.898
Without Gliptins	317	717.700	4.246	4.250	3.788

χ^2^_Yates_: The Chi-Squared with Yates' correction. ROR_025:_ The lower limit of the 95% confidence interval of the ROR. Sensitivity analysis result: Excluding cases where drugs other than DDP-4 inhibitors were suspected in the BP occurrence. a: The number of adverse events corresponding to the drug.

**Table 3:** DMAs result of data from five pharmacovigilance databases: FPVD, JADER, FEDRA, VigiBase, and FAERS

Database	a	χ^2^_Yates_	PRR	ROR	ROR_025_
FPVD	42	1867.135	65.380	67.535	47.062
JADER	392	9163.440	84.988	87.558	72.608
FEDRA	45	1627.011	69.770	71.355	47.921
VigiBase	1070	118159.250	175.504	179.430	166.362
FAERS	383	4624.373	15.408	15.470	13.916
Total	1932	87122.550	62.711	63.493	60.276

χ^2^_Yates_: The Chi-Squared with Yates' correction. ROR_025:_ The lower limit of the 95% confidence interval of the ROR. a: The number of adverse events corresponding to the drug.

**Table 4:** Association between antihyperglycemic drug exposure and bullous pemphigoid occurrence measured by disproportionality analysis

Drugs	a	χ^2^_Yates_	PRR	ROR	ROR_025_
***Alpha-glucosidase inhibitors***	12	96.178	10.738	10.770	6.105
Without Gliptins	11	91.438	11.144	11.179	6.179
***Amylin analogs***	0	NA^*^	NA^*^	NA^*^	NA^*^
Without Gliptins	0	NA^*^	NA^*^	NA^*^	NA^*^
***Incretin mimetics***	34	3.593	0.714	0.714	0.509
Without Gliptins	31	4.555	0.673	0.673	0.473
***Insulin***	142	33.700	1.642	1.643	1.389
Without Gliptins	117	12.844	1.405	1.405	1.169
***Meglitinides***	49	702.257	16.777	16.858	12.708
Without Gliptins	41	549.259	15.836	15.908	11.685
***Non-sulfonylureas***	584	2541.646	7.016	7.028	6.434
Without Gliptins	429	1462.319	5.680	5.688	5.143
***SGLT-2 inhibitors***	27	5.401	1.597	1.597	1.094
Without Gliptins	22	2.716	1.459	1.459	0.959
***Sulfonylureas***	113	163.667	3.200	3.202	2.655
Without Gliptins	82	79.936	2.637	2.638	2.120
***Thiazolidinediones***	51	1.728	1.216	1.217	0.923
Without Gliptins	32	1.571	0.790	0.790	0.558

χ^2^_Yates_: The Chi-Squared with Yates' correction. ROR_025:_ The lower limit of the 95% confidence interval of the ROR. a: The number of adverse events corresponding to the drug. NA: Not applicable due to the low number of case reports (a<3).
