# Supplementary material for: Bullous Pemphigoid and Diabetes medications: A disproportionality analysis based on the FDA Adverse Event Reporting System
Source: Int J Med Sci. 2021 Mar 3;18(9):1946–52. doi: 10.7150/ijms.55421 (PMC8040401; doi:10.7150/ijms.55421)
Supplement: Supplementary file 1 — Supplementary table 1. [file ijmsv18p1946s1.pdf]

Title: Bullous Pemphigoid and Diabetes Medications: A Disproportionality Analysis Based on the FDA Adverse Event Reporting System

Corresponding Author: Yiguo Jiang, Zaixiang Tang

Corresponding Author's Institution: The Affiliated Suzhou Science & Technology Town Hospital of Nanjing Medical University, Medical College of Soochow University

Email: Jiangyiguo0515@126.com, tangzx@suda.edu.cn

Journal name: International Journal of Medical Sciences

Order of Authors: Liting Huang, Ying Liu, Huijun Li, Weicun Huang, Ruirui Geng, Zaixiang Tang, Yiguo Jiang

Supplementary Table 1: List of drugs reported to induce bullous pemphigoid [1, 2]

Type/Drug	N	*Type*/Drug	N	*Type*/Drug	N
Antiarrythmics-antihypertensives		*Antibiotics*		*Other*	
Ca^+ channel blockers	0	Actinomycin	0	Arsenic	0
Amlodipine	34	Amoxicillin	1	Azathioprine	1
Nifedipine	2	Ampicillin	0	Clonidine	1
ACE inhibitors	0	Cephalexin	0	Denosumab	0
Captopril	0	Ciprofloxacin	0	Erlotinib	0
Enalapril	1	Chloroquine	0	Fluoxetine	9
Lisinopril	5	Dactinomycin	0	Flupenthixol	0
β-blockers	0	Levofloxacin	0	Gabapentin	8
Nadolol	0	Penicillin	0	Interleukin-2	0
Practolol	0	Rifampicin/Rifampin	0	Gold thiosulfate	0
Ramipril	16	Sulfamethoxazole	0	Ipilimumab	0

Angiotensin II antagonists	0	Trimethoprim	0	Galantamine hydrobromide	0
Losartan	1	*Vaccines*		Levetiracetam	3
Valsartan	10	Influenza	0	Mepolizumab	0
Topical		Swine flu	0	Methyldopa	0
Anthralin	0	Tetanus toxoid	0	Natalizumab	0
Benzyl benzoate	0	HZV	0	Nivolumab	4
Iodophor in adhesive bandage	0	Hexavalent combined vaccines	0	Psoralens with UVA	0
5-fluorouracil	0	*NSAID*		Thiopronin	0
Coal tar	0	Azapropazone	0	Omeprazole	13
Epinephrine	0	Celecoxib	0	Omalizumab	0
Idoxuridine	0	Diclofenac (topical)	0	Terbinafine	0
Pilocarpine	0	Ibuprofen	0	Placental extracts	0
Timolol	1	Mefenamic acid	0	Potassium iodide	0
Salicylates		Phenacetin	0	Pembrolizumab	0
Aspirin	8	*Anti TNF-α*		Risperidone	2
Sulphasalazine	0	Adalimumab	0	Rituximab	0
Salicylazosulphapyride	0	Efalizumab	0	Secukinumab	2
Salicylazosulfapyridine	0	Etanercept	1	Sulphonamide	0
Diuretics		*Antirheumatics*		Ustekinumab	0
Furosemide	12	D-penicillamine	0		
Spironolactone	15	Tiobutarit	0		

The N of supplementary Table 2 meant the bullous pemphigoid cases' number induced by the Dipeptidyl Peptidase 4 (DDP-4) Inhibitors and the combination drug (target drug reported to induce bullous pemphigoid).

To avoid overmuch events being excluded, the study only excluded the drug whose N ≥10 for the sensitivity analysis of DDP-4 Inhibitors. In a word, the study excluded

the following drugs for the sensitivity analysis: amlodipine, ramipril, valsartan, furosemide, spironolactone and omeprazole.

References

1. Stavropoulos PG, Soura E, Antoniou C. Drug-induced pemphigoid: a review of the literature. Journal of the European Academy of Dermatology and Venereology. 2014;28(9):1133-40. doi:10.1111/jdv.12366.

2. Carnovale C, Mazhar F, Arzenton E, Moretti U, Pozzi M, Mosini G et al. Bullous pemphigoid induced by dipeptidyl peptidase-4 (DPP-4) inhibitors: a pharmacovigilance-pharmacodynamic/pharmacokinetic assessment through an analysis of the vigibase(R). Expert Opin Drug Saf. 2019;18(11):1099-108. doi:10.1080/14740338.2019.1668373.
