# Supplementary material for: Bullous Pemphigoid and Diabetes medications: A disproportionality analysis based on the FDA Adverse Event Reporting System
Source: Int J Med Sci. 2021 Mar 3;18(9):1946–52. doi: 10.7150/ijms.55421 (PMC8040401; doi:10.7150/ijms.55421)
Supplement: Supplementary file 2 — Supplementary table 2. [file ijmsv18p1946s2.pdf]

Title: Bullous Pemphigoid and Diabetes Medications: A Disproportionality Analysis Based on the FDA Adverse Event Reporting System

Corresponding Author: Yiguo Jiang, Zaixiang Tang

Corresponding Author's Institution: The Affiliated Suzhou Science & Technology Town Hospital of Nanjing Medical University, Medical College of Soochow University

Email: Jiangyiguo0515@126.com, tangzx@suda.edu.cn

Journal name: International Journal of Medical Sciences

Order of Authors: Liting Huang, Ying Liu, Huijun Li, Weicun Huang, Ruirui Geng, Zaixiang Tang, Yiguo Jiang

Supplementary Table 2: Diabetes medications list

Type	Drug
alpha-glucosidase inhibitors	Acarbose, Miglitol
Amylin analogs	Pramlintide
Incretin mimetics	Albiglutide, Dulaglutide, Exenatide, Liraglutide, Lixisenatide
Insulin	
Meglitinides	Nateglinide, Repaglinide
Non-sulfonylureas	Metformin
SGLT-2 inhibitors	Canagliflozin, Dapagliflozin, Empagliflozin
Sulfonylureas	Chlorpropamide, Glimepiride, Glipizide, Glyburide, Tolazamide, Tolbutamide
Thiazolidinediones	Rosiglitazone, Pioglitazone

The above data was from https://www.drugs.com/drug-class/antidiabetic-agents.html.
