# Supplementary material for: Bullous Pemphigoid and Diabetes medications: A disproportionality analysis based on the FDA Adverse Event Reporting System
Source: Int J Med Sci. 2021 Mar 3;18(9):1946–52. doi: 10.7150/ijms.55421 (PMC8040401; doi:10.7150/ijms.55421)
Supplement: Supplementary file 3 — Supplementary table 3. [file ijmsv18p1946s3.pdf]

Title: Bullous Pemphigoid and Diabetes Medications: A Disproportionality Analysis Based on the FDA Adverse Event Reporting System

Corresponding Author: Yiguo Jiang, Zaixiang Tang

Corresponding Author's Institution: The Affiliated Suzhou Science & Technology Town Hospital of Nanjing Medical University, Medical College of Soochow University

Email: Jiangyiguo0515@126.com, tangzx@suda.edu.cn

Journal name: International Journal of Medical Sciences

Order of Authors: Liting Huang, Ying Liu, Huijun Li, Weicun Huang, Ruirui Geng, Zaixiang Tang, Yiguo Jiang

Supplementary Table 3: 2×2 table

	Reaction(s) of interest	All other reactions	Total
Drug of interest	a	b	a+b
All other drugs	c	d	c+d
Total	a+c	b+d	a+b+c+d

ROR= $\frac{ad}{bc}$, 95% CI= $e^{\ln(ROR)\pm 1.96\sqrt{(\frac{1}{a}+\frac{1}{b}+\frac{1}{c}+\frac{1}{d})}}$

PRR= $\frac{a\times(b+d)}{b\times(a+c)}$, $\chi^2_{Yates}=\frac{(a+b+c+d)\times\left(\left|(a\times d)-(b\times c)\right|-\frac{(a+b+c+d)}{2}\right)^2}{(a+b)\times(c+d)\times(a+c)\times(b+d)}$
